# Seroprevalence and risk factor associated with respiratory viral pathogens in dual-purpose cattle of Aguachica, Rio de Oro, and La Gloria municipalities in Cesar department, Colombia

**DOI:** 10.14202/vetworld.2019.951-958

**Published:** 2019-07-04

**Authors:** Juan Carlos Pinilla León, Wilson Diaz, María Cristina Vasquez, Julio Cesar Tobón, Alfredo Sánchez, Diego Ortiz

**Affiliations:** 1 Department of Veterinary Medicine, University of Santander, Faculty of Exact, Natural and Agricultural Sciences, Animal Science Research Group, Bucaramanga, Colombia; 2 Department of Bacteriology and Clinical Laboratory, University of Santander, Faculty of Health Sciences, Research Group in Clinical Management, Bucaramanga, Colombia; 3 Vecol S.A., Bogotá, Colombia; 4 Agrosavia, Bogotá, Colombia

**Keywords:** bovine herpesvirus type 1, bovine parainfluenza-3 virus, bovine respiratory syncytial virus, bovine viral diarrhea virus, seroprevalence, viral diseases

## Abstract

**Aim:**

The research was conducted to determine the seroprevalence and risk factor associated with respiratory viral pathogens in dual-purpose cattle of Aguachica, Rio de Oro and La Gloria municipalities in Cesar department, Colombia.

**Materials and Methods:**

The seroprevalence study was done from the random sampling (n=1000) of blood collected from 29 dual-purpose herds, located in three municipalities (Aguachica, Rio de Oro, and La Gloria) of Cesar department. The presence of antibodies against bovine herpesvirus type 1 (BHV-1), bovine respiratory syncytial virus (BRSV), bovine viral diarrhea virus (BVDV), and bovine parainfluenza-3 virus (BPI-3V) in the samples was detected by indirect enzyme-linked immunosorbent assay. Epidemiological data were obtained using a questionnaire administered to the owner or manager of each herd.

**Results:**

The overall highest seroprevalence was observed for BHV-1 (94.7%), followed by BRSV (98.6%), BVDV (35.2%), and BPI-3V (47.1%). Regarding the seroprevalence by municipalities, there was a statistical association (p<0.05) for BVDV; however, for BRSV, BHV-1, and BPI-3V, no statistical association was found (p>0.05) between seropositive values and the municipalities, indicating that animal was seropositive in similar proportions in the three municipalities. Female sex and older animals (>24 months) were a significant risk factor for BHV-1 and BPI-3V infection. Regarding the clinical signs, there was a statistical association (p<0.05) between the seropositive values of BVDV and most of clinical signs observed, except for abortion.

**Conclusion:**

This research confirms the high seroprevalence of the respiratory viral pathogens in nonvaccinated cattle within the study areas. Therefore, appropriate sanitary management practices and routine vaccination programs should be adopted to reduce the seroprevalence of these infectious agents.

## Introduction

Bovine respiratory disease complex (BRDC) is a major cause of economic losses in the cattle industry worldwide. The most important viral agent include bovine herpesvirus type 1 (BHV-1), bovine viral diarrhea virus (BVDV), bovine respiratory syncytial virus (BRSV), and bovine parainfluenza-3 virus (BPI-3V) [1]. BRSV, belonging to the genus *Pneumovirus* within the family *Paramyxoviridae*, and is one of the most important causes of lower respiratory tract infections in calves [2]; however, adult animals with subclinical infection are the main source of infection, since reinfections are common in the herds. It is highly prevalent in cattle, with a significant economic impact as the most important viral cause of BRDC worldwide [3]. BVDV is a *Pestivirus* from the family *Flaviviridae* [4], which affects the digestive, respiratory, and reproductive systems in different production animals [5]. Clinical signs include pyrexia, diarrhea, reduced production, and highly morbid disease but cause low mortality of infected animals [6]. Infectious bovine rhinotracheitis (IBR) is an important infectious disease of domestic and wild cattle caused by BHV-1. This virus is a member of genus *Varicellovirus*, which belongs to the *Herpesviridae* family. Clinical signs infection includes symptoms of inflammatory reactions in respiratory, genital tracts, abortion, and neurological disorders [7]. Betancur *et al*. [8] found a statistical association between seropositive animals for BHV-1 with respect sex and age in Colombia, while Ochoa *et al*. [9] reported higher infection in cows older than 5 years of age. BPI-3V is in the genus *Respirovirus* of the family *Paramyxoviridae* [10], which cause serious economic losses in small and large ruminants. Clinical disease is usually mild, with symptoms of fever, nasal discharge, and cough [11]. Betancur *et al*. [12] reported a statistical association between seroprevalence values for BPI-3V and age groups.

Aguachica, Rio de Oro, and La Gloria municipalities are located in Cesar department, which, in turn, is located in the Northeast of Colombia, and is very important agricultural and fish raising region, being the dual-purpose cattle husbandry one of the most important agricultural components of the regional economy, with a participation of 8% in the cattle national inventory. According to the National Agricultural Institute [13], the state has a population of 1,305,984 heads of cattle, being 30% located in the three municipalities.

Information about the prevalence of these viral pathogens is available from several countries in which these diseases have been reported [14-20]. Nevertheless, there is very little epidemiological information on viral pathogens in cattle, mainly in the Northeast region of Colombia. Therefore, the present study was conducted to estimate the seroprevalence of respiratory viral pathogens in dual-purpose cattle and evaluate risk factors in the municipalities of Aguachica, Rio de Oro, and La Gloria in the department of Cesar.

## Materials and Methods

### Ethical approval

This research was approved by the Institutional Ethical Committee of the University of Santander and VECOL, Colombia.

### Study area

**Figure-1:**
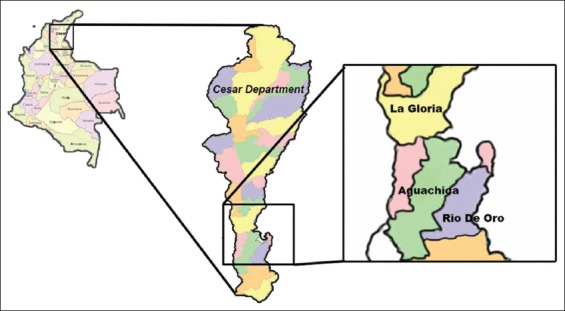
The political map of Colombia (left) and the study area (middle and right) with the three municipalities (La Gloria, Aguachica, and Rio de Oro). (Source: http://mapadecolombia.org/mapa-de-colombia-con-sus-departamentos)

The study was conducted between January and April 2017 in the municipalities of Aguachica (8°182’243’’N 73°362’553’’O), Rio de Oro (8°172’303’’N 73°232’143’’O), and La Gloria (8°37’07’’N 73°48’09’’O) in the Department of Cesar, Colombia (Figure-1). Bioclimatic characteristics of the region are a mean annual temperature of 28°C, with little weather variation along the year. Altitude is between 65 and 800 masl and mean annual rainfall is 1835 mm, with well-defined dry and wet periods [21].

### Study design and sampling

Random sampling, descriptive, and transversal were designed. Twenty-nine dual-purpose herds were randomly selected according to the base of vaccination records of the [13]. In each examined herds, samples were randomly collected. According to ICA [13], the cattle population census for the three municipalities was 391,000 heads: Using the formula for known populations [22], with an expected prevalence of 50%, a confidence level of 95%, and associated maximum error of 3%, “n” of 1000 blood samples was determined. For serum, 5 ml whole blood was collected from the coccygeal venipuncture in a sterile vacutainer tube without ethylenediaminetetraacetic acid. The serum was separated and clarified by centrifugation at 8000 rpm for 10 min and stored at −20°C until the tests were performed. The total samples were proportionally distributed according to the total number of bovine present in each herd examined. Included animals showing clinical signs and healthy animals, belonged to the dual-purpose type, with ages between 7 months and 13 years. The following two age groups were conformed: <24 months and >24 months. Epidemiological data on potential risk factors were obtained using a questionnaire administered to the owner or manager of each herd at the time the blood samples were collected. According to this information, none of the cattle herds were vaccinated against BHV-1, BRSV, BVDV, and BPI-3. Other data gathered in the questionnaire were included in the analysis: Municipality, herd size (small: 50-200 and large: 201-500 animals), clinical signs (no and yes), age group of the animal (≤24 and 24 months old), and to sex, i.e. male and females. The category for “herd size” was based on the median herd size data.

### Serological analysis

Serum samples were analyzed by indirect enzyme-linked immunosorbent assays (ELISA) according to the manufacturer’s instructions. This ELISA was done using INgezim antibody test kits for BVDV, BRSV, and BHV-1 (Ingenasa, Madrid, Spain), and Monoscreen ELISA BPI3 (Bio-X Diagnostics, Rochefort, Belgium). The results were read in a microplate photometer, where the optical density (OD) was measured at 450 nm. The cutoff was calculated as A = OD (corrected negative control). Nevertheless, results obtained were expressed as positive and negative based on the manufacturer’s recommended cutoff value for each pathogen.

### Statistical analysis

Prevalence was determined by dividing the number of positive animals between the total animal of the sampled population. The results obtained were analyzed by the Chi-square test (χ^2^) to determine the statistical association between the variables, and the odds ratio (OR) was calculated to determine the probability of risk of the analyzed factors. Calculations were made using the SPSS Statistics for Windows, (IBM, USA) version 21.0 [23].

## Results

A total of 1000 serum samples were screened from 29 herds located in three municipalities of Cesar state. Overall, seroprevalence for BVDV in the Aguachica, Rio de Oro, and La Gloria municipalities was 35.2%; while for BRSV, BHV-1, and BPI-3V were 98.6%, 94.7%, and 47.1%, respectively. These values were analyzed by the Chi-square test and according to the results obtained, there was statistical association (p<0.05) between the seropositive values of BVDV in the three municipalities: About 25.5% (164/643) in Aguachica, 54.4% (37/68) in La Gloria, and 52.2% (151/289) in Rio de Oro. According to these results, the seroprevalence for BVDV is present in different proportions in the three municipalities of the Cesar department, Colombia. Nevertheless, for BRSV, BHV-1, and BPI-3V, no statistical association was found (p>0.05) between seropositive values and the municipalities, indicating that the seroprevalence is present in similar proportions in the three municipalities (Table-1). Regarding the herd size (small and large), all the examined herds were positive for antibodies against BHV-1, BRSV, and BPI-3V; however, for BVDV 86.2% (25/29) resulted positive. According to these results, viral pathogens circulate widely in the herds with different size of the evaluated municipalities.

**Table 1:** Comparison between seropositive values for respiratory viral pathogens in cattle from Aguachica, La Gloria, and Rio de Oro municipalities, Cesar department, Colombia.

Municipality	n	Herds	SP for BVDV(%)	SP for BRSV(%)	SP for BHV-1(%)	SP for BPI-3V(%)
Aguachica	643	19	164 (25.5)	635 (98.8)	608 (94.6)	303 (47.1)
La Gloria	68	2	37 (54.4)	68 (100)	63 (92.6)	32 (47.1)
Rio de Oro	289	8	151 (52.2)	283 (97.9)	276 (95.5)	136 (47.1)
Total	1000	29	352 (35.2)	986 (98.6)	947 (94.7)	471 (47.1)
Chi-square(χ^2^)			74.3	2.03	0.96	0.00
p-value			0.000	0.36	0.61	1

Statistically significant (p<0.05). BPI-3V=Bovine parainfluenza-3 virus, SP=Seroprevalence, BVDV=Bovine viral diarrhea virus, BRSV=Bovine respiratory syncytial virus, BHV-1=Bovine herpesvirus type1

Table-2 shows the comparison between percent positive for the viral pathogens and the age of the animals. These values were analyzed by Chi-square test, and according to with the results obtained, there was no statistical association (p>0.05) between the seropositive values of BVDV, BRSV, and BPI-3V with respect to age groups. In contrast, there was a statistical association (p<0.05) between positive percent for BHV-1 and the age of the animals. Young calves were no tested for any respiratory viral pathogens.

**Table 2:** Comparison between seropositive values for viral pathogens in cattle according to the age of the animals.

Age	n	SP for BVDV(%)	SP for BRSV(%)	SP for BHV-1(%)	SP for BPI-3V(%)
<24 months	23	10 (43.5)	23 (100)	19 (82.6)	9 (39.1)
>24 months	977	342 (35)	963 (98.6	928 (95)	462 (47.3)
Total	1000	352 (35.2)	986 (98.6)	947 (94.7)	471 (47.1)
χ^2^(Chi-square)		0.7	0.3	6.8	5.4
p-value		0.4	0.5	0.009	0.01

Statistically significant (p<0.05). BPI-3V=Bovine parainfluenza-3 virus, SP=Seroprevalence, BVDV=Bovine viral diarrhea virus, BRSV=Bovine respiratory syncytial virus, BHV-1=Bovine herpesvirus type 1

Table-3 shows the frequency of clinical signs observed in the animals tested. The retention of placenta presented the highest frequency (75.1%), followed by abortion (56.8%), fever (56.8%), and diarrhea (55.7%). The diseased animals showed temperature ranges above 40°C and the abortions pattern was observed in any trimester of the gestation. The comparison between percent positive for the viral pathogens with the presence or absence of clinical signs is shown in Table-3. These values were analyzed by Chi-square test and according to the results obtained, there was a statistical association (p<0.05) between the seropositive values of BVDV and the most of clinical signs observed, except for abortion (p>0.05). In contrast, there was no statistical association (p>0.05) between positive percent for BHV-1, BRSV, and BPI-3V with the most of clinical signs, except for vulvovaginitis and conjunctivitis in BHV-1 and BPI-3V, respectively. All herds infected with BRSV showing similar clinical signs. In the present study, other infectious pathogens were not studied; however, the sensitivity of the ELISA test is high, which allows to estimate the seroprevalence of these viral pathogens in the dual-purpose cattle breeds.

**Table 3:** Frequency (%) and the association between the clinical signs with seropositive values of the respiratory viral pathogens in the tested animals (n=1000).

Clinical signs	Frequency(%)	Category	χ^2^(Chi-square); p -value			

			BHV-1	BRSV	BPI-3V	BVDV
Retention of placenta	75.1	Yes	(0.5); p=0.4	(0.8); p=0.4	(2.2); p=0.1	(48.8); p=0.00
		No				
Abortion	56.8	Yes	(0.07); p=0.7	(0.2); p=0.6	(1.1); p=0.3	(1.4); p=0.2
		No				
Fever	56.8	Yes	(1.4); p=0.2	(1.1); p=0.3	(0.03); p=0.8	(58.1); p=0.00
		No				
Diarrhea	55.7	Yes	(1.6); p=0.2	(3.1); p=0.08	(2.0); p=0.1	(94.1); p=0.00
		No				
Conjunctivitis	38.5	Yes	(0.01); p=0.9	(1.7); p=0.2	(5.7); p=0.01	(47.2); p=0.00
		No				
Respiratory	34.9	Yes	(0.5); p=0.4	(0.2); p=0.6	(1.2); p=0.2	(111); p=0.00
		No				
Fetal death	31.7	Yes	(2.1); p=0.1	(0.06); p=0.8	(0.1); p=0.7	(76.3); p=0.00
		No				
Vulvovaginitis	13.7	Yes	(5.5); p=0.02	(2.2); p=0.1	(2.5); p=0.1	(8.09); p=0.004
		No				

Statistically significant (p<0.05). BPI-3V=Bovine parainfluenza-3 virus, BVDV=Bovine viral diarrhea virus, BRSV=Bovine respiratory syncytial virus, BHV-1=Bovine herpesvirus type1

In the logistic regression analysis, the significant risk factors were sex and age group for BHV-1 and BPI-3V and the municipality for BVDV (Table-4). Females showed 3.2 (OR=3.2, IC95%=0.9-11.5) and 3.6 (OR=3.6, IC95%=1.2-10.9) times higher risk of infection by BHV-1 and BPI-3V than males. Regarding the age, cows older than 24 months showed 3.9 and 3.5 times probability for infection of BHV-1 and BPI-3V, respectively, than younger cows. La Gloria and Rio de Oro municipalities showed 3.5 and 3.2 times higher probability of infection for BVDV than Aguachica municipality. There was no statistical significance between the pathogens with respect to the herd size (p>0.05).

**Table 4:** Results of logistic regression analysis for BHV-1, BVDV, BRSV, and BPI-3V infection in cattle.

Risk factor	BHV-1				BVDV				BRSV				BPI-3V			
			
	*b*	OR	95%CI	p-value	*b*	OR	95%CI	p-value	*b*	OR	95%CI	p-value	*b*	OR	95%CI	p-value
Municipality																
Aguachica	-	1	-	-	-	1	-		-	1	-	-	-	1	-	-
Rio de Oro	−0.2	0.8	0.6-2.3	0.5	−1.1	3.2	2.4-4.3	0.00	0.4	0.6	0.2-1.7	0.3	0.01	1	0.7-1.3	0.9
La Gloria	0.32	1.3	0.2-1.9	0.5	−1.3	3.5	2.1-5.8	0.00	−16.5	0	0	0.9	−0.02	1	0.6-1.6	0.9
Sex																
Male	-	1	-	-	-	1	-		-	1	-	-	-	1	-	-
Female	−1.1	3.2	0.9-11	0.04	−0.04	0.8	0.3-2	0.6	16.9	0	0	0.9	−1.3	3.6	1.2-10.9	0.01
Age(months)																
<24	-	1	-	-	-	1	-		-	1	-	-	-	1	-	-
>24	−1.3	3.9	1.3-12		0.8	0.7	0.3-1.6	0.4	16.9	0	0	0.9	−0.3	3.5	0.7-16.8	0.01
Herd size(heads)																
50-200	-	1	-	-	-	1			-	1	-	-	-	1	-	-
201-500	−0.3	0.7	0.4-1.2		0.08	1.2	0.8-1.4	0.3	−0.5	0.4	0.1-1.4	0.3	0.02	1	0.7-1.3	0.9

Statistically significant (p<0.05). *b*=Coefficient b. OR=Odds ratio. CI=Confidence interval. 1=Reference category. BPI-3V=Bovine parainfluenza-3 virus, BVDV=Bovine viral diarrhea virus, BRSV=Bovine respiratory syncytial virus, BHV-1=Bovine herpesvirus type1

## Discussion

The viral pathogens of cattle in this research represent a major health problem in the worldwide and are important since they cause an increase in morbidity and high economic losses in the herd. This is the first epidemiological study on viral pathogens carried out in the department of Cesar, Colombia, to determinate seroprevalence.

### BRSV

Our results revealed high BRSV seroprevalence (98.6%) in the three explored municipalities that indicate most adult cattle have been exposed to this pathogen. The herd seroprevalence of BRSV (100%) found in this research is consistent with published data of Solis-Calderon *et al*. [14], Saa *et al*. [18], and Carbonero *et al*. [24], who reported a herd prevalence of 90.8% (Mexico), 91.3% (Ecuador), and 95.8% (Argentina), respectively. However, these results differ from those reported by Obando *et al*. [17] Contreras and Parra [25], who found lower seroprevalence values in other studies. The individual seroprevalence of BRSV (98.6%) agrees with the findings of Saa *et al*. [18] who reported 80.4% of seroprevalence in herds of Ecuador. This result also agrees with those of Betancur *et al*. [19] and Betancur *et al*. [20] who found 13% and 31% of seroprevalence in dairy cows and calves, respectively, in herds of Montería state, Colombia.

Nevertheless, these results differ from those published by Carbonero *et al*. [24], who reported 46.6% of seroprevalence in Argentina. The results obtained demonstrate that BRSV is widespread among animals and dual-purpose cattle herds in Cesar department. Probably, after the initial infection occurs in some animals, the virus is rapidly spread throughout the animals, probably by aerosols, particularly in herds without prior exposure to the virus, increasing seropositivity [17]. The several herds in Colombia are not being vaccinated against BRSV and result from this research demonstrates that this virus circulates among the animals and herds from the three municipalities. It would be important to include BRSV in vaccination programs with the aim of controlling infection in this region.

Regarding the age, BRSV infection was observed in both age groups in this research. Although the analysis was not done in younger animals, as reported in the literature, the clinical disease is more frequent in calves [26]. This seroreactivity in adult animals suggests possible reinfections during the course of their life. The result obtained in this research agrees with those reported by Betancur *et al*. [19], who found no statistical association between infection and age group. Nevertheless, the results obtained differ from those published by Bidokhti *et al*. [27], who found statistical differences with respect to the age of the infected animals. They demonstrate that after infection with BRSV, the animals will remain seropositive for several years. The older cows were seropositive while the younger cows were seronegative, i.e., there had been no virus circulating for several years. In this study, municipality, sex, and herd size were not a significant risk factor (Table-4). Regarding the clinical signs, animals with respiratory problems (34.9%) and conjunctivitis (38.5%) were found (Table-3). However, there was no statistical association (p>0.05) between seroprevalence values and respiratory signs in tested animals. These results are due to BRSV, which is observed in any age group, but infections that result in severe clinical disease are typically observed in calves [26]. Nevertheless, there was no sampling in calves in this research.

### BVDV

BVDV has been reported to be the virus most commonly isolated from pneumonic lungs of cattle. This pathogens has a global distribution and impacts the respiratory and reproductive performance of the animals, resulting in significant financial losses. Three hundred and fifty-two (35.2%) of the 1000 serum samples were positive for BVDV antibody (Table-1). This result agrees with those by Betancur *et al*. [8] who reported similar values of seroprevalence for BVDV in cattle of Monteria state, Colombia. In addition, the results obtained agree with the previously reported prevalence (35-90%) in various studies in Australia, USA, Canada, Colombia, and Argentina [28]. However, the results obtained in this study differ with those published by García-Chaparro *et al*. [15], who reported 10.34% of seroprevalence for BVDV in a herd with the reproductive problem in Cundinamarca, Colombia. These results also differ with those published by Giraldo *et al*. [29] and Corro *et al*. [30], who reported 58% and 55.43% of seroprevalence in the bovine herd from Colombia and Venezuela.

Regarding the region studied, there were significant differences between the seroprevalence of BVDV in the three municipalities (p<0.05). Rio de Oro (OR=3.2) and La Gloria (OR=3.5) municipalities showed higher seroprevalence and were an important risk factor for BVDV, which suggest the presence of persistently infected animals in these municipalities. This result agrees with those reported by Segura-Correa *et al*. [31], who demonstrate statistical differences between the rural districts in North Eastern of Mexico. The variation of seroprevalence in different regions may be due to differences in cattle population age, cattle density, herd size, housing systems, biosecurity, disease control measures, management practices, and type of breeding of the animals, which in general could be important risk factors in transmission and persistence of BVDV [31,32].

Regarding the age, the result obtained differs with those published by Corro *et al*. [30] and Segura-Correa *et al*. [33], who reported higher seroprevalence values in animals older than 24 months of age. In our study, the similar seroprevalence of BVDV observed between the two age groups suggests the dissemination of persistently infected animals in the herds studied. In this study, sex, age, and herd size were not a significant risk factor (Table-4).

With respect to clinical signs, there was a statistical association (p<0.05) between the seropositive values of BVDV and most of clinical signs observed, except for abortion. There was a high frequency in cattle having a previous history of retention of the placenta (75.1%). In this regard, 475 cows of 1000 animals had retention of placenta, with 47.5% BVDV seropositive and this indicates that there was a significant difference (p<0.05) between two groups of categories (Table-3). These findings in this study may be due to other causal agents of retention of placenta in the cattle such as bacterial, parasitic, or any other viral, causing these findings in this study. There was a high frequency in cattle having fever and diarrhea (56.8% and 55.7%), respectively. Diarrhea and fever had a statistical association (p<0.05) with the seroprevalence of BVDV in the study animals. BVDV causes diarrhea, enteritis, and atrophy of the intestinal villi [34]; however, it is not possible to explain the statistical association found in this study because other causes inducing diarrhea and fever in animals were not studied.

### Bovine parainfluenza virus type 3 (BPI-3V)

BPI-3V sometimes cause severe disease as a single agent and can predispose the animal to bacterial infections of the lung [35]. Our results revealed high BPI-3V seroprevalence (47.1%) in the three explored municipalities that indicate most adult cattle have been exposed to this pathogen. These results agree with those by Carbonero *et al*. [14], who found high seroprevalence values in cattle of Yucatan, Mexico. However, the results obtained in this study differ with those published by Betancur *et al*. [12], who reported lower seroprevalence values (13.5%) in cattle from Monteria, Colombia. The high seroprevalence of BPI-3V found in this research is in agreement with the ubiquitous nature of the virus and with its worldwide distribution [36]. In this research, the seroprevalence was higher in the age group of >24 months of age (Table-4). This age group was a significant risk factor for BPI-3V transmission (OR=3.5). Possibly, due to the presence of some stress factor in these animals that favors reinfections with or without respiratory signs. In adults, especially BPI-3V, it is subclinical unless it is part of concomitant infections with other viruses and bacteria such as *Pasteurella multocida*, *Mannheimia haemolytica*, *Mycoplasma* spp., and immunosuppressive factors [37]. With regard to the clinical signs, conjunctivitis had a statistical association with the BPI-3V seroprevalence values, and regarding sex, female was a significant risk factor for BPI-3V infection (OR=3.6). This result differs with those published by Betancurt *et al*. [12], who found no statistical association between BPI-3V infection and sex.

### BHV-1

Our results in Table-1 demonstrate a high level of exposure to this virus in the municipalities studied with seroprevalence of 94.7%. These results agree with those by Betancur *et al*. [8] who reported high values of seroprevalence for BHV-1 in herds of Monteria municipality, Colombia. However, these results differ with those by Ochoa *et al*. and [9] Guarino *et al*. [38], who reported 35.6% and 37% seroprevalence in cattle of Boyaca department, Colombia and Uruguay, respectively. With regard to the clinical signs, vulvovaginitis had a statistical association with the BHV-1 seroprevalence values, and sex and age were significant risk factors for the viral infection. The age wise seroprevalence to BHV-1 infection denoted that there was an increase in the occurrence of the infection as age advances, and seroprevalence was low in the young animals. In this research, it is revealed that the seroprevalence of BHV-1 in animals over 24 months of age was found to be higher than the lower age groups (<24 months). For this reason, the seroprevalence of BHV-1 in cows may reflect the proportion of BHV-1 carriers because, after a primary infection, the virus stays latent in neural ganglions that innervate genital or respiratory mucosae and maybe re-excreted on immuno-suppressive stimuli, such stress after shipment and calving [36]. With respect, the sex, the high prevalence in female might be attributed to the use of infected bulls for natural service and infected semen for artificial insemination [39]. The fact that animals were not vaccinated and that all age-groups had high seroprevalence indicates that the viral pathogens respiratory are naturally circulating in this population. So is urgently needed to establish measures for the epidemiological control and prevention of these diseases with the aim to decrease their incidence. Therefore, vaccination of animals could be considered. The distribution of virus and risk factors identification is important to establish prevention and control programs against economically important diseases such as BPI-3V, BRSV, BVDV, and BHV-1.

## Conclusion

This research confirms the high seroprevalence of the viral pathogens respiratory in nonvaccinated cattle within the study areas. Therefore, appropriate sanitary management practices and routine vaccination programs should be adopted to reduce the seroprevalence of these infectious agents.

## Authors’ Contributions

AS and DO conceived and designed the research. JCT conducted the sample collection. JCPL, WD, MCV, and JCT processed samples in the Laboratory of Bacteriology. JCPL carried out the data analysis and writing of the manuscript. All the authors read and approved the submitted version of the manuscript.
